# Integration of enzyme kinetic models and isotopomer distribution analysis for studies of *in situ *cell operation

**DOI:** 10.1186/1471-2202-7-S1-S7

**Published:** 2006-10-30

**Authors:** Vitaly A Selivanov, Tatiana Sukhomlin, Josep J Centelles, Paul WN Lee, Marta Cascante

**Affiliations:** 1 Department of Biochemistry and Molecular Biology, Faculty of Chemistry, Marti i Franques, 1, 08028 Barcelona, Spain; 2 CERQT-Parc Cientific de Barcelona, Barcelona, Spain; 3 Institute of Theoretical and Experimental Biophysics, Pushchino, 142290, Russia; 4 Department of Pediatrics, Harbor-UCLA Medical Center, Research and Education Institute, Torrance, CA 90502, USA

## Abstract

A current trend in neuroscience research is the use of stable isotope tracers in order to address metabolic processes *in vivo*. The tracers produce a huge number of metabolite forms that differ according to the number and position of labeled isotopes in the carbon skeleton (isotopomers) and such a large variety makes the analysis of isotopomer data highly complex. On the other hand, this multiplicity of forms does provide sufficient information to address cell operation *in vivo*. By the end of last millennium, a number of tools have been developed for estimation of metabolic flux profile from any possible isotopomer distribution data. However, although well elaborated, these tools were limited to steady state analysis, and the obtained set of fluxes remained disconnected from their biochemical context. In this review we focus on a new numerical analytical approach that integrates kinetic and metabolic flux analysis. The related computational algorithm estimates the dynamic flux based on the time-dependent distribution of all possible isotopomers of metabolic pathway intermediates that are generated from a labeled substrate. The new algorithm connects specific tracer data with enzyme kinetic characteristics, thereby extending the amount of data available for analysis: it uses enzyme kinetic data to estimate the flux profile, and *vice versa*, for the kinetic analysis it uses *in vivo *tracer data to reveal the biochemical basis of the estimated metabolic fluxes.

## Introduction: application of stable isotope tracer data in neuro -biology and -medicine

**Figure 1:**
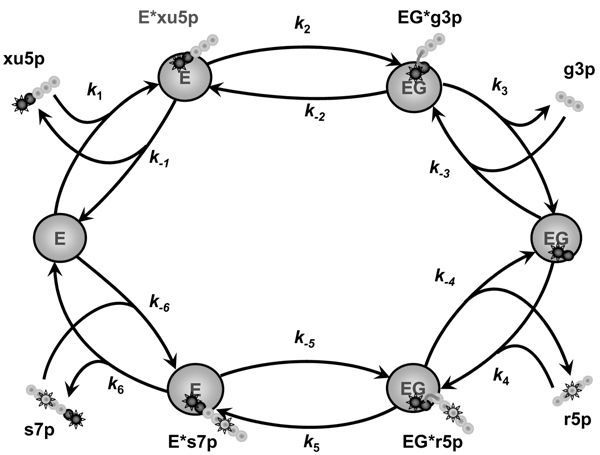
An example of isotope exchange in one of the reactions of non-oxidative pentose phosphate pathway catalyzed by transketolase: xu5p + r5p <-> g3p + s7p. The catalytic cycle consists of a series of reversible elementary steps: binding of donor substrate (xu5p) and formation (k_1_, k_-1_) of a covalent enzyme-substrate complex (E*xu5p); splitting (k_2_, k_-2_) of donor substrate and formation of a covalently bound intermediate (the α-carbanion of α, β-dihydroxyethyl-ThDP, the so-called 'active glycolaldehyde') and an aldose (g3p); both are localized in the active site of the enzyme (EG*g3p). This complex dissociates (k_3_, k_-3_) into the complex of the enzyme with active glycolaldehyde (EG) and the first product, free aldose (g3p). In the second half-reaction, active glycolaldehyde interacts with the other aldose (r5p) available in the reaction mixture (k_4_, k_-4_). The new ketose (s7p) is released from the enzyme-substrate complex after passing through the same reaction steps in reverse order (k_5_, k_-5 _and k_6_, k_-6_). Large circles represent the protein molecule, while small linked circles represent the carbon skeleton of the metabolites. Two dark circles represent the part of substrate attached to the enzyme during whole catalytic cycle and to be transferred between ketoses. The gray circles are the parts released after ketose splitting. Stared circles are labeled carbon atoms. The scheme presents, as an example, formation of non-labeled g3p and double labeled s7p from xu5p labeled in first position and r5p labeled in third position.

Metabolic networks of living cells produce the intricate redistribution of carbon skeleton atoms of substrates. If these substrates are artificially labeled by stable isotopes (such as ^13^C) at specific positions, the reorganization of carbon skeleton becomes measurable and its quantification provides insight to the respective metabolic reactions. Figure 1 shows an example of isotope exchange performed by one of the reaction catalyzed by transketolase (TK) in the non-oxidative pentose phosphate pathway (PPP). Interconnection of several isotope exchange reactions creates in each metabolite a variety of forms, which differ by the number and positions of ^13^C isotopes (^13^C isotopomers). A given set of metabolic fluxes produces a specific distribution of isotopomer fractions, and consequently, the isotopomer distribution indicates the underlying set of fluxes.

Two techniques for isotopomer detection were used to estimate metabolic fluxes *in situ: *nuclear magnetic resonance (NMR) and mass spectrometry (MS). The different kinds of data that can be obtained from NMR measurements were classified by Mollney *et al *[1]. One-dimensional ^1^H NMR measurement can provide positional ^13^C enrichment, i.e. fraction of molecules with label in specific carbon position in a molecule [2,3]; multiple peaks of one dimensional ^13^C NMR reveal groups of isotopomers [4]. Two-dimensional ^1^H-^13^C NMR essentially allows quantification of individual isotopomer fractions [5,6]. MS provides information on the fractions of different mass isotopomers [7,8], i.e. groups of isotopomers with a fixed number of labels regardless their positions. It detects fewer isotopomers than NMR but is usually more sensitive.

Stable isotope detection is now used for the metabolic flux profile estimation in various areas of cell and tissue biology, and neuroscience is not an exception. Implementations of the *in vivo *NMR measurements to study brain function were described in the recent review article [9]. Notably, ^13^C NMR studies recently showed the significance of glutamate release and recycling between neurons and glia. The neurotransmitter pool, which previously was considered to be small and metabolically inactive, appeared to be included in glutamine-glutamate recycling, the major neuronal metabolic pathway. The activity associated with glutamate neurotransmission was found to be linearly dependent upon glucose oxidation, and this supports the molecular model of stoichiometric coupling between glutamate neurotransmission and functional glucose oxidation. Thus, the considered here models of central glucose metabolism are clearly of great interest to the field of neuroscience.

Since ^13^C is known to be harmless, it has been used in human subjects [10], particularly for mapping the spatial localization of metabolites using NMR spectroscopic imaging [3,11]. This method, designed for clinical use, has been applied as a diagnostic tool in pediatric and adult brain disorders [12,13]. A non-invasive ^13^C magnetic resonance spectroscopy technique allowed the synthesis rate of *N*-acetyl-1-aspartate to be determined in patients with Canavan disease [14,15]. It is also widely used in neuroscience to study brain metabolism in animals [16,17]. Bixel et al [18] revealed an interesting application of this method by using it to define all products of leucine metabolism in cultured astroglial cells.

While most of the results described above were obtained by the qualitative comparison of NMR measurements, their quantitative analysis would offer much more profound insights into cell processes. A tool able to estimate metabolic fluxes would reveal the dynamic characteristics of cellular phenotype under specific conditions, thus complementing genomic and proteomic methods, which only reveal static characteristics of the cell. The specific tools for quantitative metabolic flux profile analysis from measured isotopomer distribution data are described next.

## Current status of isotopomer distribution analysis

Very approximate estimation of metabolic fluxes could be provided by implementation even formulas derived using simplifications such as an assumption that the fluxes are unidirectional [19-22]. Such formulas were applied to the analysis of enrichment in specific carbon position or mass isotopomers. Although such analysis is easy to perform, it is incomplete, and as we have found (unpublished data), the simplifications could lead to unreliable estimates. More reliable tools that supplement data acquisition normally include the following: (i) a mathematical model, which simulates the distribution of isotopomers, (ii) algorithms used to fit respective experimental pattern and thus evaluate the matching flux profile, and (iii) statistical analysis of the best fit parameters.

Different levels of complexity could be implemented in mathematical models for the isotopomer distribution analysis. Katz and Wood [23] were the first who estimated the metabolic fluxes by solving the mass balance equations for the positional enrichment of metabolites. However, this approach was only appropriate for a small number of fluxes that, in addition, were unidirectional. Consideration of real bidirectional fluxes increased a number of unknown parameters rendering the systems of equations underdetermined. This disadvantage demanded more advanced analytical applications, which would be able to analyze more experimental data in order to unambiguously determine the parameters. Mason and Rothman [24] described the basic principles of simple model construction for the analysis of some selected positional enrichment together with executing a "classical" kinetic model that evaluated the time course of metabolite concentrations using the available kinetic information. The idea to complement kinetic models with isotopomer distribution data and, in the same time, to link isotopomer analysis with kinetic data increased the reliability and informative outcome of both approaches. However, the described method for isotopomer analysis cannot account for all the information provided by NMR experiments. For any given molecule containing n carbon atoms, it only filters a maximum of n enrichments at selected carbon positions, while the total number of all isotopomers is 2^n ^and all of them could be important for the estimation of the metabolic flux profile. Thus a part of information contained in the NMR data is not used for the analysis. In fact, the NMR measurements, as it is mentioned in the introduction, could provide the relative concentrations of individual isotopomers or at least the sum of those that contain a specific label pattern.

The comprehensive analysis of mass isotopomer data obtained by MS also demands that all possible individual isotopomers are computed, because every mass isotopomer is the sum of several individual isotopomer concentrations and each component of the sum could be produced in the different way.

According to the total number of isotopomers, the calculation of their fractions requires solving 2^n ^equations for the substance consisted of n carbons. In this way even for the model description of all isotopomers formation in glycolysis and PPP the algorithm must construct (it is practically impossible to write such a huge number of equations by hand) and solve several hundred equations.

Schmidt *et al*. [25] developed a simulation algorithm for the computation of all possible isotopomers. This algorithm of isotopomer mapping matrices, evolved from the earlier approach of atom mapping matrices [2,26], allowed to construct and solve automatically hundreds of equations that describe all isotopomers transformation according to the specific reaction mechanisms. The iterative procedure, which was used for the solution, in fact provided the evaluation of time course for the relaxation of each isotopomer fraction to steady state. This method made it possible to simulate all isotopomers distribution for the given set of metabolic fluxes, while the best fit to the experimental distribution pointed to the set compatible with the measured distribution. However, the presence of large exchange fluxes caused severe instability of numerical solution or convergence problems [27], which restricted the application of this method.

Wiechert et al [27] found an elegant way to overcome the problem of instability by reformulating isotopomer balance equations into cumomer balance equations. The term cumomer (from cumulative isotopomers) designates a sum of isotopomers with fixed positions of label (a 1-cumomer fraction is a sum of the positional enrichments). Such reformulation of the equations in terms of cumomers simplified the task and made it possible to obtain the solution in one step based on matrix calculus. The equations formulated in terms of cumomer fractions can be solved explicitly as a cascade of linear systems, evaluating the cumomer fractions one by one starting from the 0-cumomer fraction. Then the cumomer fractions can be transformed back into isotopomer fractions.

This method was well elaborated and all the history of its development from analysis of positional enrichment experiments was well documented [27-30]. A computer program based on the cumomer-balance method performs the complete isotopomer analysis and finds the flux profile by experimental isotopomer distribution fitting [27]. It is easy to work with this program; user just needs to write in symbolic form the reactions designed for analysis marking the respective re-formations of carbon skeleton. Using this input, the program automatically constructs isotopomer balances, transforms to cumomer analysis, solves the equations, and displays the results in the desired form. Creation of such a tool seemed to solve eventually the problems of isotopomer analysis.

However, there are at least three reasons to develop one more approach to isotopomer analysis. First, although the above described algorithm overcomes the problem of instability of iterative numerical solution, it is completely restricted by stationary flux analysis thus leaving without any examination the available time course of label distribution. In fact, the stability of solution could be controlled without losing the advantage of testing the time course of isotopomer accumulation, which could be more informative than steady state analysis.

Second, all the above approaches to the computation of isotopomer transformations consider fluxes as independent variables. This was noted as an advantage of the method because such an analysis did not need any assumptions regarding the biochemical basics of considered fluxes [27]. However, this could be an advantage only if the experimental pattern of label distribution is sufficient for evaluation of a unique true set of fluxes. If the amount of information is insufficient, additional data are necessary for the unambiguous evaluation of flux profile. In this case the known kinetic characteristics of analyzed enzyme reactions and results of classical kinetic modeling could provide such additional necessary information.

Third, even if the analysis reveals the fluxes taking them as independent, the fluxes remain disconnected from the detailed mechanisms of the catalysis and regulation considered in kinetics models, thus the biochemical reasons for the observed behavior remain unclear. In this case, the use of kinetic modeling could also solve this problem. Long era of classical biochemistry developed a number of kinetic models of complex systems that use known characteristics of enzyme catalysis and regulation. These parameters could be employed in metabolic flux analysis, providing the necessary additional information. An excellent example is a model of erythrocyte central metabolism [31], which includes kinetic models of all involved enzymes with known regulatory and catalytic mechanisms and kinetic constants, verified by numerous experiments.

On the other hand, the kinetic models of complex systems, analyzing the experimentally observed cellular functions as a result of operation of many regulated processes, include many parameters and therefore, like the flux analysis, also suffer from insufficiency of experimental data. Moreover, the kinetic models normally include characteristics of enzymatic reactions obtained *in vitro*, and such data cannot always be used for the interpretation of *in vivo *experiments. The use of *in vivo *tracer data would animate the old classical district of kinetic studies.

In this situation integration of kinetic modeling with complete isotopomer analysis would provide:

- for kinetic study of *in vivo *cell operation the new area of tracer data, which are necessary for understanding the organization and regulation of the processes in living cells and applicability of classical *in vitro *information;

- for metabolic flux analysis the additional information to restrict the number of acceptable sets of metabolic fluxes by the ones that are compatible not only with a given pattern of isotopomer distribution, but also with the data of previous biochemical studies.

The way of such integration is described next.

## Algorithms for integrated kinetic and metabolic flux analysis

As MFA is a commonly accepted acronym for metabolic flux analysis based on isotopomer data, the tool proposed by Selivanov et al [32,33] designed for integrated kinetic and metabolic flux analysis is abbreviated here as IKMFA.

IKMFA was designed to possess the following characteristics:

1. To be compatible with any kinetic model of central metabolism, so that the MFA part accepts the total fluxes and metabolite concentrations predetermined by the kinetic model constituting the first part of the analytic software.

2. To use the total fluxes and metabolite concentrations, obtained from the kinetic model, for estimation of the time courses and distribution of all possible isotopomers.

3. To use fitting of the experimental time course, or/and steady state isotopomer distribution or/and global metabolite concentrations for the estimation of both fluxes and parameters of the kinetic model used.

**Figure 2:**
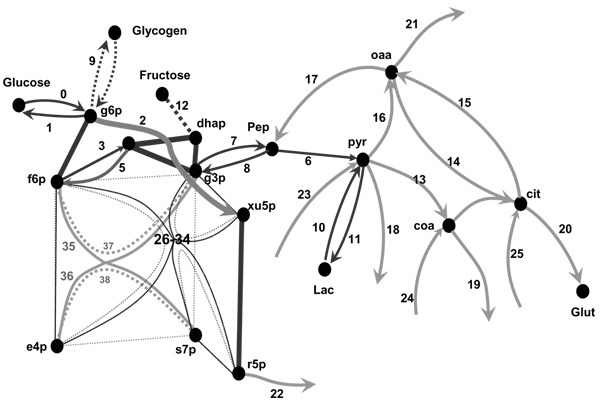
Scheme of the metabolic reactions simulated in the model comprising glycolysis and gluconeogenesis, PPP, TCA cycle and anaplerotic reactions. The nodes represent metabolites, and solid lines are reactions. Reaction 1 and 0 describe glucose exchange with the medium; reaction 2 is the oxidative branch of PPC (g6p → (r5p<->r15p<->x5p)); 3–11 belong to glycolytic or gluconeogenesis pathways (PFK, aldolase, Fl, 6Pase, g3p conversion through GPDH reaction); 12 is fructose input, 13 is pyruvate decarboxylation, 14–15 are simplified representation of the TCA cycle, 16–17 are anaplerotic reactions, 18–25 are the in- and out-fluxes connecting the considered part with the rest of metabolic network, 26–34 and 35–38 are respectively transketolase and transaldolase isotope exchange fluxes described in the text. Thick edges indicate fast equilibrium between the connected nodes, catalyzed by EP and RPI, PGI, TPI.

These characteristics render IKMFA able to implement the detailed tissue-specific kinetic mechanisms of the enzyme reactions to describe the fluxes. In other words this approach builds a complete isotopomer analysis on the top of a kinetic model. Used for the analysis of isotopomer distribution data, the kinetic mechanisms, validated by all available kinetic models, could be examined for the *in vivo *conditions. This links the estimated metabolic fluxes with molecular and kinetic mechanisms of the metabolic pathways, i.e. with their biochemical basis. There are no limits in respect to the expressions for reaction rates, catalysis could be described according to the kinetic mechanism of any levels of complexity as it is accepted in kinetic modeling. An application of IKMFA for analysis of complex metabolic system that connects kinetic models of central metabolism with MFA has been considered elsewhere [33]. This metabolic system is schematically represented in the Figure 2.

As indicated above, the first step of analysis is the solution of ordinary differential equations (ODEs), which describe the total concentration change as the sum of the production rate of the given metabolite minus the rates of its consumption.

In principle any ODE solver could serve in the kinetic part of the analysis. Since the software was written in programming language "C++", a compatible ODE solver was chosen. The existed source "C++" libraries, such as "Numerical recipes in C++",  or the C++ class library 'ODE++' of Milde (2003),  were appropriate solvers. The metabolite concentration obtained from the ODEs solution become initial values of non-labeled isotopomers of internal metabolites in the following step of analysis. Other isotopomers are initially set to 0, except the outside metabolites, which have the initial distribution according to that added experimentally. Fluxes, also obtained from the solution of ODEs, are used in the second part to simulate the respective reactions between the isotopomers (Figure 2).

In spite of evident similarity between the first step of IKMFA and ordinary kinetic model execution, there is an essential difference between them. Kinetic models normally relate one enzyme reaction to the net flux as the difference between the forward and reverse fluxes, because only net fluxes define changes in the calculated total metabolite concentrations. In contrast, the first step of IKMFA needs to compute the forward and reverse fluxes separately and also some additional fluxes, which also serve as an input for the subsequent MFA. Definitions of such additional fluxes and a way to compute them are described next.

## Definitions and algorithm for evaluation of all isotope-exchange fluxes

A classical example of a variety of fluxes is the non-oxidative PPP, the most problematic metabolic part related to numerous isotope-exchange reactions catalysed by TK and TA [34,35]. TK, thiamine diphosphate (ThDP)-dependent enzyme, catalyzes cleavage of a carbon-carbon bond and reversibly transfers a two-carbon fragment (glycolaldehyde) from a ketose-phosphate donor to an aldose- phosphate acceptor, forming new ketose- and aldose-phosphates. TA catalysis operates via another mechanism involving a Schiff base formed directly between the enzyme and the substrate, and, as a result, a three-carbon fragment (dihydroxyacetone) is transferred from a ketose-phosphate donor to an aldose-phosphate acceptor. However, a similar scheme could represent the steps of TA catalysis and TK catalysis is therefore considered here as representative of both enzymes. Although only two TK reactions are widely accepted to take place in PPP,

r5p + x5p <-> s7p + g3p     (1)

**Figure 3:**
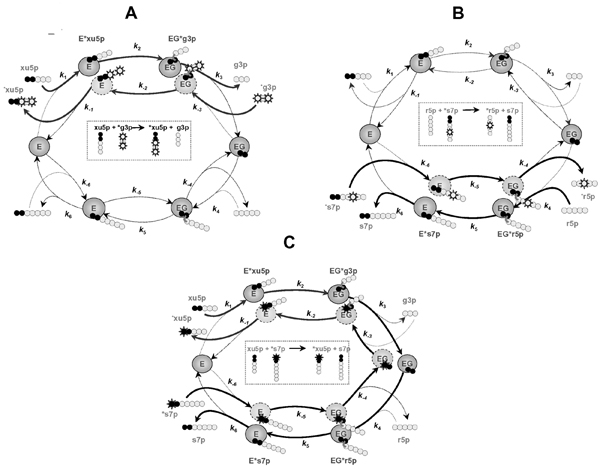
Various isotope exchange fluxes created in the TK-catalyzed reaction: xu5p + r5p <-> g3p + s7p. Designations are the same as in Figure 1. **A**. Isotope exchange between xu5p and g3p in the presence of labeled g3p results in labeling of xu5p. This exchange flux could be calculated as follows. Forward flux of the last *n*-2 atoms of ketose-substrate to the pool of aldose-product (e.g., x5p → g3p) implies delivering the x5p atoms through the three steps (x5p + E → E*x5p → EG*g3p → EG + g3p). The intermediates E*x5p and EG*g3p contain aldose fragments originated from two sources, either x5p or g3p, and the respective fractions of isotopomers are specified by the relative values of the elementary rates. Specifically, the rate of delivery of x5p atoms into g3p is a part of the rate *v*_3 _(*v*_i _is a unitary rate corresponding to the rate constant *k*_i_); it is proportional to the content of carbon atoms originated from x5p in EG*g3p. The proportionality constant or fraction of x5p atoms in EG*g3p (*P*^x1^_EGg_, where the superscript x1 denotes the last carbons originated from x5p, and the subscript EGg denotes the form EG*g3p) depends on the fraction of former x5p atoms in E*x5p, that partly consists also of former g3p atoms that enter via the reactions whose rates are *v*_-2 _and *v*_-3_; thus it is expressed as a ratio of the input of the donor atoms from E*x5p (whose fraction is *P*^x1^_Ex_) to the total input to EGa_1_: *P*^x1^_EGg _= (*v*_2_*P*^x1 ^_Ex_)/(*v*_2 _+ *v*_-3_).     (f1) The proportion of atoms in E*x5p that originated from x5p (*p*^x1^_Ex_) in turn is given by the ratio of influx of this kind of atom to the total influx into the compound Ec_1 _at steady state: *P*^x1 ^_Ex _= (*v*_1 _+ *v*_-2_*P*^x1^_EGg_)/(*v*_1 _+ *v*_-2_).     (f2) Solving Eqs fl and f2 yields the expression: *P*^x1^_EGg _= (*v*_1 _*v*_2_)/(*v*_-2 _*v*_-3 _+ *v*_-3 _*v*_1 _+ *v*_1 _*v*_2_).     (f3) The flux of former x5p atoms into g3p, *v*_xg_, where the subscript xg denotes the x5p->g3p direction, is given by *v*_xg _= *v*_3 _*P*^x1^_EGg _= (*v*_3 _*v*_1 _*v*_2_)/(*v*_-2 _*v*_-3 _+ *v*_-3 _*v*_1 _+ *v*_1 _*v*_2_).     (f4) Equation f4 gives the rate of forward delivery of the last *n*-2 atoms in x5p to g3p, expressed by using the unitary rates. Since these atoms can originate only from either x5p or g3p, the fraction of atoms originating from g3p is expressed as *P*^g^_Ex _= 1 - *p*^x1^_Ex_. and the reverse flux of the aldose (g3p) to the ketose pool (x5p) can be described similarly to Eq. f4 as *v*_gx _= *v*_-1 _*P*^g^_Ex_,     (f5) **B**. Isotope exchange between s7p and r5p in the presence of labeled s7p results in labeling the r5p. The exchange of atoms between s7p and r5p can be described in the same way as it is done in **A**. **C**. Isotope exchange between s7p and x5p in the presence of labeled s7p results in labeling of x5p. Forward flux (*v*_xs_) of the first two atoms of x5p to a second ketose/donor, s7p, implies delivery of the atoms through six steps (x5p → E*x5p → EG*g3p → EG → EG*r5p → E*s7p → s7p). This is a part of the rate of s7p production (*v*_6_) and it is proportional to the fraction of former x5p carbon atoms in E*s7p, namely *P*^xf^_Es_, where the superscript xf denotes that the first part of the molecule originates from x5p. This proportion is determined similarly to that described above, i.e. by solving the five equations for the fractions of atoms that originated from x5p in all the species (similar to the Eqns fl and f2). The reverse flux (*v*_sx_) of the first two atoms of s7p to x5p could be described in the same way. Thus the following fluxes of the carbon skeleton parts are expressed through the same elementary steps of the catalytic mechanism: *v*_xg_: x5p -> g3p *v*_gx_: g3p -> x5p *v*_xs_: x5p -> s7p *v*_sx_: s7p -> x5p *v*_sr_: s7p -> r5p *v*_rs_: r5p -> s7p The difference between forward and reverse fluxes of isotope exchange between all pairs of pools is the same and corresponds to the net flux: *v*_xg _- *v*_gx _= *v*_xs _- *v*_sx _= *v*_rs _- *v*_sr _= *v*_net _    (f6) It follows from (f6) *v*_xg _- *v*_xs _= *v*_gx _- *v*_sx_, and *v*_sr _- *v*_sx _= *v*_rs _- *v*_xs _    (f7) The whole reaction related to exchange between x5p and s7p expressed by the fuxes *v*_xs _and *v*_sx _is accompanied by the exchange inside half-reactions, i.e. between x5p and g3p, and also between s7p and r5p. These exchanges in fact constitute a part of the fluxes *v*_xg _and *v*_sr _deduced above and the differences (f7) describe the "pure" exchange between ketose and the product of its splitting, which is the same in both directions. Taking into account equality of the "pure" exchanges expressed by equations (f7), the four fluxes define all of the isotope exchanges associated with the considered TK reaction: - forward flux x5p->s7p (*v*_xs_) - reverse flux s7p->x5p (*v*_sx_) - pure exchange x5p<->g3p (*v*_xg _- *v*_xs_) - pure exchange s7p<->r5p (*v*_sr _- *v*_sx_) The above fluxes could be expressed through the elementary rates, as exemplified by Equation f4. The elementary rates, in turn, could be expressed through the elementary rate constants and substrate and product concentrations using, for instance, King and Altman algorithm (as described e.g. in [48]). Thus, all TK fluxes are considered not as independent but as interrelated through the elementary rate constants, which could be determined in independent experiments as described elsewhere [33].

e4p + x5p <-> f6p + g3p,     (2)

this enzyme catalyzes in fact much more isotope-exchange reactions [34,35]. Using as an example the first of two above reactions (also shown in Figure 1), Figure 3 illustrates all possible isotope exchange fluxes related with this TK reaction.

The reversible steps associated with the ping-pong mechanism of TK reaction involve exchange (i) between the ketose substrate and product of its cleavage, or, in the present example, between xylulose-5-phosphate (xu5p) and glyceraldehyde-3-phosphate (g3p) shown in Figure 3A and also between sedoheptulose-7-phosphate (s7p) and ribose-5-phosphate (r5p) shown in Figure 3B, and (ii) between two ketoses (xu5p and s7p, Figure 3C). Thus, six isotope exchange fluxes are associated with one TK reaction. As shown previously [32] all of them are essential for distribution of the label and should be taken into account for correct estimation of metabolic fluxes. These fluxes could be expressed through the rate constants of elementary steps as the legend to Figure 3 describes in details.

In fact, forward and reverse transfer of the first two carbons between the x5p and s7p are coupled with transfer of the three last carbons of x5p to g3p pool and back, and also the five last carbons of s7p to r5p pool and back. Therefore, transfer between ketose and product of its cleavage could be presented as consisted of two parts, one of which is coupled with transfer between two ketoses and some another, additional part. The legend to Figure 3 explains in details that these additional parts are equal in forward and reverse directions. Taking this into account, one should compute (in the way shown in the legend to Figure 3) four different isotope transfers related with just one TK reaction (1):

- xu5p->s7p accompanied by the transfer xu5p->g3p and r5p->s7p

- s7p->xu5p accompanied by the transfer s7p->r5p and g3p->xu5p

- additional transfer xu5p<->g3p the same in both directions

- additional transfer s7p<->r5p the same in both directions.

**Figure 4:**
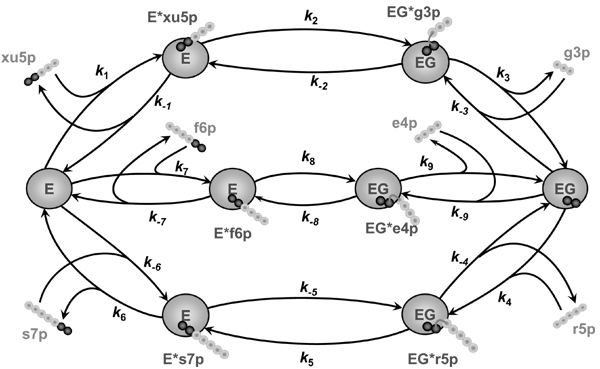
Scheme of all the TK reactions accounting for competition between them. Designations are the same as in Figure 1. The reactions start with reversible binding of the free enzyme to ketose (with the elementary rate constants *k*_1_, *k*_-1_, *k*_7_, *k*_-7_, *k*_6_, *k*_-6_) and formation of the covalent enzyme-substrate complex followed by its splitting (*k*_2_, *k*_-2_, *k*_8_, *k*_-8_, *k*_5_, *k*_-5_) and formation of the covalently bound intermediate G ('active glycolaldehyde') and aldose, both localized in the active site of the enzyme. The split complex dissociates (*k*_3_, *k*_-3_, *k*_9_, *k*_-9_, *k*_4_, *k*_-4_) into the enzyme bound with active glycolaldehyde (EG) and the free molecule of aldose. Nine different isotope exchange fluxes are associated with these reactions, as explored in more detail in Figure 3. xu5p + E → Exu5p → EGg3p  EG  EGrSp → Es7p → E + s7p xu5p + E ← Exu5p ← EGg3p  EG  EGrSp ← ES ← E + s7p xu5p + E → Exu5p → EGg3p  EG  EGe4p → Ef6p → E + f6p xu5p + E ← Exu5p ← EGg3p  EG  EGe4p ← Ef6p ← E + f6p s7p + E → Es7p → EGr5p  EG  EGe4p → Ef6p → E + f6p s7p + E ← Es7p ← EGr5p  EG  EGe4p ← Ef6p ← E + f6p xu5p+E↔Exu5p↔EGg3p↔EG+g3p f6p+E↔Ef6p↔EGe4p↔EG+e4p s7p+E↔Es7p↔EGrSp↔EG+r5p

Presence of different substrates in the intracellular volume further complicates the situation. Figure 4 shows three reactions catalyzed by TK *in vivo*, which compete for the same enzyme. In this situation the number of isotope exchange fluxes related to the TK reactions increases, but all of them can be expressed through the elementary rate constants in the way similar to that indicated in the Figure 3 legend. The following isotope-exchange fluxes related to the TK reactions shown in the Figure 4 should be accounted for subsequent analysis in the way similar to that described in the legend of Figure 3 for one separated reaction:

- xu5p->s7p accompanied by the transfer xu5p->g3p and r5p->s7p;

- s7p->xu5p accompanied by the transfer s7p->r5p and g3p->xu5p;

- xu5p->f6p accompanied by the transfer xu5p->g3p and e4p->f6p;

- f6p->xu5p accompanied by the transfer f6p -> e4p and g3p -> xu5p;

- s7p->f6p accompanied by the transfer s7p->r5p and e4p->f6p;

- f6p->s7p accompanied by the transfer f6p->e4p and r5p->s7p;

- additional transfer xu5p<->g3p the same in both directions;

- additional transfer s7p<->r5p the same in both directions;

- additional transfer e4p<->f6p the same in both directions;

Expressed through the elementary rate constants such isotope exchange fluxes are evaluated for all the enzymes in the course of executing the kinetic model. The obtained values are used in the second step of analysis, namely in simulation of isotopomer distribution, which is done in the way similar to that accepted in MFA (e.g. in [27]). The respective algorithm is described next.

## Reactions between isotopomers

To interpret the result of isotope redistribution in metabolites of a metabolic pathway all the possible reactions between isotopomers are simulated at this step of analysis. An algorithm of simulation of isotopomer distribution, which is present below, is in principle similar to that described elsewhere [25], though it is implemented by different means. In contrast to the earlier algorithms [25], here the total fluxes are taken from the previous step of analysis, where they are expressed as functions of total concentrations of metabolites and effectors according to the reaction mechanisms. Moreover, the program calculates the real concentrations and time courses of isotopomers and then defines fractions corresponding to the analyzed experimental data.

For isotopomer designation we use a binary notation for the ^13^C and ^12^C atoms as it is helpful in optimization of references to a specific isotopomer. Since each carbon atom of a molecule can exist in one of the two states: labeled (marked as '1') or unlabeled ('0'), each metabolite in the model can be represented by an array of 2^n ^of possible isotopomers, where n is the number of carbon atoms in the molecule. Each isotopomer in the model is represented as binary numbers; its digits correspond to the carbon atoms in a molecule (3 digits for trioses, 4 for erythrose, etc.). A '1' or a '0' in certain position in a string signifies that corresponding carbon atom is labeled or unlabeled. For instance, all isotopomers for glyceraldehyde-3-phosphate (g3p) are:

000, 001, 010, 01l, 100, 101, 110, 111

This representation of isotopomers as successive integer numbers is very convenient because the model uses it as references to the respective position in the existing array of concentrations of all isotopomers, different for each metabolite. This ordering of the isotopomers in the array allows optimization of referring the isotopomer products for any isotopomer substrate related to the considered reactions, as it is explained below. For instance, the reaction (xu5p + r5p ↔ s7p + g3p) between arbitrarily chosen isotopomers of x5p (10011) and r5p (10000) produces the following isotopomers of s7p and g3p:

10011(xu5p) + 10000(r5p) ↔ 1010000(s7p) + 011(g3p)     (3)

This reaction could be easily simulated by manipulations with binary numbers, *viz*., the product of scission of the ketose substrate of n carbons will be a product of bitwise AND of the respective binary number with 2^n-2^-1, in the above example (10011 AND 111) = 011. To define the reference number of the produced new ketose, the reference number of initial ketose should be shifted n-2 positions right, and then (if m is number of carbons in the aldose substrate) m positions left. The bitwise OR operation performed for the obtained number and reference number of aldose substrate produces the resulted ketose isotopomer. In the above example, right shift of 10011 gives 10, its left shift gives 1000000 and (1000000 OR 10000) gives 1010000. These operations are fast and allow the reference number of isotopomer products to be defined for each pair of isotopomer-substrates.

Determination of the reference numbers of products for each pair of substrates is the basis of the optimization. Then the change of concentrations according to the succession of reaction is calculated. Assuming that all isotopomers have the equal affinity, the rate of reaction between pair of isotopomers is proportional to their concentrations, while the sum of reaction rates for all isotopomers gives the global metabolic flux calculated also at the previous, kinetic, step. If, for instance, Vf is the global forward flux for the TK reaction between X5P and R5P, the flux for this reaction between isotopomers i and j (V3f_ij_) would be expressed as follows:

Vf_ij _= Vf × [X5P_i_] × [R5P_j_]/([X5P_tot_] × [R5P_tot_])     (4)

Here, the indices i and j refer to the concentrations of the respective isotopomers and the index 'tot' refers to the total concentration of the metabolite, as calculated in the kinetic model. In this way, the first part, ODE solving, is linked to the second part that computes the label distribution: the kinetic model calculates global fluxes and concentrations and defines the values (as in the above example V3f/([X5P_tot_] x [R5P_tot_])), which are used to get the fluxes in reactions between isotopomers (as in Equation (4)). During the small time interval *dt *the TK reaction (3) considered as an example consumes the amount *dt *× V3f*ij *of each of the isotopomers *i *and *j *and this value is subtracted from the concentrations of respective isotopomers.

[X5P_*i*_]_*t*+*dt *_= [X5P_*i*_]_*t *_- *dt *× Vf_*ij*_

[R5P*j*]_*t*+*dt *_= [R5P_*j*_]_*t *_- *dt *× Vf_*ij *_    (5)

Here, the indices *t *and *t + dt *indicate the time of the simulated process. As usual for numerical solution of differential equations, this could be acceptable approximation if the time step *dt *is so small that consumption during one step is small compared to the amount of these isotopomers; practically, the value for *dt *is taken so that its further decrease does not affect the solution. The program adds the same amount to the concentrations of reaction products, which numbers (*ra *and *rd*) are defined from *i *and *j *as described above.

[GAP_*ra*_]_*t*+*dt *_= [GAP_*ra*_]_*t *_+ *dt *× Vf_*ij*_

[S7P_*rd*_]_*t*+*dt *_= [S7P_*rd*_]_*t *_+ *dt *× Vf_*ij *_    (6)

The above algorithm is present in order to illustrate the main principles of TK reaction simulation between a pair of isotopomers according to the Equations 5 and 6. However this algorithm is not optimized in the best way. To simulate the reaction between n isotopomers of ketose and m isotopomers of aldose it is necessary to perform n × m cycles of such calculations. It could be optimized by the imaginary separation of the whole reaction into two steps corresponding to the aldose and ketose products formation. First scission of all ketose isotopomers could be simulated, producing respective isotopomers of aldose product according to the scheme x5p->g3p; this demands just n recalculations of x5p and g3p concentrations. Then the rest two-carbon fragment (for TK) with only 4 different possible combinations of label interacts with isotopomers of aldose substrate (C_2_+r5p->s7p). This demands 4 × m recalculations of r5p and s7p for one direction of the reaction. In this way the whole description of the TK reaction needs only 2×(n+4×m) recalculations of substrate-product pairs instead of n × m recalculations of four substances according to the above algorithm.

Analysis of experimental data starts from execution of the kinetic model simulating time course of metabolite concentrations and fluxes, which then are used in the second step of simulation of corresponding labeled isotopomer distribution. Experimental data fitting finds the flux profile and kinetic parameters compatible with the analyzed data. This is described next.

## Fitting algorithm

A combination of kinetic modeling with isotopomer distribution analysis allows combining also the respective experimental data, which can be analysed. Moreover, IKMFA expands the usual steady state isotopomer analysis to the non-steady state conditions. The following kinds of experimental data could be subjects of fitting:

- Measured rate of production of various metabolites in cell cultures under different conditions of incubation and intracellular concentration of some metabolites;

- Total metabolite concentrations;

- Distribution of labeled atoms such as ^13^C isotopes in metabolites (^13^C isotopomers), when the label was added with some of the substrates.

Optimization of a merit function in a multidimensional space of parameters is well elaborated and usually does not represent a problem [36,37]. However, in our particular case, two kinds of computational problems arise. First, if the merit function (χ^2^, the sum of the squares of the vertical distances of the experimental points from the calculated curve divided by the standard deviation) is defined from the numerical solution of differential equations, abrupt change of parameters demanded by the applied fitting algorithm could render the system stiff and induce failure of ODE solver. Second, if the total metabolite concentrations are not measured, the merit function does not account for them, so that the best fit sometimes corresponds to evidently unreal concentrations.

The first problem was partly solved by our approach of two-step solution, when the isotopomer analysis comes after the differential equations that describe changes in total metabolite concentrations are solved. In the first step a small number of equations for total concentrations are to be solved and at this step the solution is usually robust. A number of numerical methods for ODE solution are implemented, in particular the Bulirsch-Stoer method [37] could be recommended. If the problem of stiffness nevertheless arises it could be solved using implicit or semi-implicit methods e.g. using the Bader and Deuflhard discretization of Bulirsch-Stoer method [37]. The problem of stiffness often arises in the second step when much more equations for isotopomers have to be solved. The methods for stiff ODEs will be implemented for the solution of the huge systems for isotopomers, however, even the use of the steady state metabolite concentrations, known from overall the solution, as initial values for unlabeled isotopomers improves the robustness of the solution.

Another part of the stiffness problem in fact was solved when, to address the second problem (of unreal best-fit concentrations), we introduced the threshold for χ^2 ^change, dependent on metabolite concentrations. According to the used Powel's algorithm for the local decrease of χ^2 ^value, the program changed parameters one by one until the minimum was reached in each direction. To avoid long descent in almost flat valley, if a change in χ^2 ^was lower than threshold, the program switched to another direction in the space of parameters. If some metabolite concentration has reached its critical value, the program automatically increased the threshold for χ^2 ^change if the parameter change increased the critical concentration, but decreased the threshold to 0 if the parameter change induced also a decrease of critical concentration. These rules let to control the range of concentrations, and made the solution of ODEs more stable. After termination of descent in the space of parameters, the program make a step uphill by random change of a parameter as is supposed by Simulated Annealing algorithm, and then repeats the downhill descent. All the successful steps of parameter change are saved, so that if the program comes into the area of stiffness and the solver fails, the procedure could be started again from the last successful step after the necessary correction.

After the program finds the minimum of χ^2 ^and completes the fitting procedure, it can determine the set of parameters most relevant to the fit using singular value decomposition of the second derivative matrix of χ^2 ^with respect the parameters (Hessian matrix). Square roots of the diagonal elements of the inverse of Hessian matrix provide the standard deviation for the essential parameters. The present in our website instruction describes the way of using this feature of the program.

Thus, fitting algorithm accepts different kinds of data and estimates not only compatible set of metabolic fluxes but also parameters of enzyme reactions and their regulation, providing insight to the biochemical underground of the particular set of metabolic fluxes.

## Prime results and perspective

The advantages of implementation of comprehensive enzyme kinetic mechanisms (e.g. as explored in the Figure 3 and shown in Figure 4) in metabolic flux analysis were examined in [33], where the two levels of profundity in flux analysis were compared. When the fluxes created in TK reaction were considered as independent variables, as was commonly accepted currently in metabolic flux analysis, they were compatible with the experimental r5p and lactate label distribution in cancer cell line HT29. However, the set of isotope-exchange fluxes obtained in this way was not unique and was found to be incompatible with the TK reaction mechanism. The implementation of dependency between all TK fluxes through the unitary rates, as was accepted in classical enzyme kinetic analysis, restricted the area of possible estimates thus making the analysis more reliable.

On the other hand, such integrated analysis brought a new area of isotopomer distribution data to the kinetic study of enzyme operation *in vivo*. Fitting isotopomer distribution directly accessing the kinetic constants provided insight to the enzyme kinetic mechanisms that operate *in vivo*. Some of the kinetic constants obtained from *in vitro *study were corrected based on the analysis of *in vivo *isotopomer distribution. The ability to accumulate the information from different sources and to check its compatibility makes this approach scalable into analytical tool which filters and orders available data, as well as evaluates metabolic fluxes compatible with maximum of available information.

The analysis of tracer data considered here focuses mainly on central metabolic pathways that, according to the recent review [9], are stoichiometrically coupled with glutamate neurotransmission. In this view a major limitation in the interpretation of tracer data is the poor definition of relationship between neuronal activity and neuroenergetic processes supported by glucose metabolism [38-40]. The term neuronal activity applies to a spectrum of energy requiring processes including action potential propagation, neurotransmitter release and uptake, vesicular recycling, and maintenance of membrane potentials [41]. All of these processes are involved in short-term neuronal information encoding and the relative distribution of energy among them remains to be determined. The contribution of the different classes of neurons in a cortical region to the overall energy consumption remains also unclear. To address these questions the flux analysis based on tracer data should be coupled with the comprehensive models of central metabolism interactions with neuronal activity; this assumes further development of IKMFA towards higher complexity of the kinetic model underlying the flux analysis. Such a model could include different cellular compartments or even consist of partial differential equations describing the diffusion of components in intracellular volume. The requirement for multi-compartment analysis of label distribution data follows, in part, from a series of experiments where even qualitative analysis of metabolite labeling pointed out to the existence of different pools of pyruvate in neurons and astrocytes [42-44]. The existence of non-mixed pools of soluble substance in cytosole currently seems surprising. Using a multi-compartment model coupled with isotopomer analysis for the analysis of such data would allow other possible hypotheses to be excluded and would provide quantitative information regarding the relationship between the pools.

Although the data on the existence of different intracellular compartments not separated by membrane structures are not widely accepted, they appear in various areas of cell biology and deserve thorough analysis. Analysis of the behavior of ATP-sensitive K+ channel, located in sarcolemma revealed large differences in ATP levels at different distance from the sarcolemma [45,46]. Similarly high diffusion limitations in the membrane vicinity were found for cAMP, as follows from the study of cAMP gated channels [47]. Understanding real cellular processes requires integrated analysis of various data relating to the real multi-compartment intracellular space with restricted diffusion limitation for metabolites. The approach described here has an ability to develop into a tool that can be used in such an integrative analysis. An understanding of regulatory mechanisms as an addition to the reliable estimations of metabolic fluxes will increase outcome of stable isotope tracer analysis, which is already in clinical use.

## Abbreviations

cit, citrate; dhap, dihydroxyacetone phosphate; e4p, erythrose-4-phosphate; g6p, glucose-6-phosphate; g3p, glyceraldehyde-3-phosphate; f6p, fructose-6-phosphate; glu, glutamate; lac, lactate; oaa, oxaloacetate; pep, phosphoenolpyruvate; PPP, pentose phosphate pathway; pyr, pyruvate; r5p, ribose-5-phosphate; s7p, sedoheptulose-7-phosphate; TA, transaldolase; TK, transketolase; xu5p, xylulose-5-phosphate; NMR, nuclear magnetic resonance; MS, mass spectrometry; MFA, metabolic flux analysis.
